# Supplementary material for: Machine Learning Accelerates Crystallization for Structure Determination
Source: Angew Chem Int Ed Engl. 2026 May 4;65(25):e1218503. doi: 10.1002/anie.1218503 (PMC13266958; doi:10.1002/anie.1218503)

checkCIF/PLATON report

Structure factors have been supplied for datablock(s) lcz068

THIS REPORT IS FOR GUIDANCE ONLY. IF USED AS PART OF A REVIEW PROCEDURE FOR PUBLICATION, IT SHOULD NOT REPLACE THE EXPERTISE OF AN EXPERIENCED CRYSTALLOGRAPHIC REFEREE.

No syntax errors found. CIF dictionary Interpreting this report

Datablock: lcz068

Bond precision: C-C = 0.0130 A Wavelength=1.54184

Cell:	a=13.3641(1)	b=14.7506(1)	c=32.2331(3)
	alpha=90	beta=90	gamma=90
Temperature:	100 K		

	Calculated	Reported
Volume	6354.06(9)	6354.06(9)
Space group	P 21 21 21	P 21 21 21
Hall group	P 2ac 2ab	P 2ac 2ab
Moiety formula	C49 H30 Ag6 F36 N12 O3	2(C15 H3 Ag3 F18 N6), C19 H24 O3
Sum formula	C49 H30 Ag6 F36 N12 O3	C49 H30 Ag6 F36 N12 O3
Mr	2166.07	2166.07
Dx,g cm-3	2.264	2.264
Z	4	4
Mu (mm-1)	16.024	16.024
F000	4152.0	4152.0
F000’	4172.23	
h,k,lmax	17,18,41	16,18,40
Nref	13696[7528]	12673
Tmin,Tmax	0.102,0.125	0.694,1.000
Tmin’	0.023	

Correction method= # Reported T Limits: Tmin=0.694 Tmax=1.000
AbsCorr = MULTI-SCAN

Data completeness= 1.68/0.93 Theta(max)= 78.732

R(reflections)= 0.0347(11955) wR2(reflections)= 0.0923(12673)

S = 1.130 Npar= 958

The following ALERTS were generated. Each ALERT has the format
test-name_ALERT_alert-type_alert-level.
Click on the hyperlinks for more details of the test.

Alert level B

```
PLAT780_ALERT_1_B Coordinates do not Form a Properly Connected Set     Please Do !
```

Author Response: The alert is due to a large number of co-crystallized molecules in the which sometimes do not show as a connected set. This is acceptable from a crystallographic point of view.

Alert level C

```
PLAT042_ALERT_1_C Calc. and Reported MoietyFormula Strings  Differ     Please Check
              Calc: C49 H30 Ag6 F36 N12 O3
              Rep.: 2(C15 H3 Ag3 F18 N6), C19 H24 O3
PLAT090_ALERT_3_C Poor Data / Parameter Ratio (Zmax > 18) ........       7.64 Note
PLAT213_ALERT_2_C Atom F01K            has ADP max/min Ratio .....        3.1 prolat
PLAT342_ALERT_3_C Low Bond Precision on  C-C Bonds ..............    0.01296 Ang.
PLAT911_ALERT_3_C Missing FCF Refl Between Thmin & STh/L=    0.600          3 Report
                0 17  9,   0 17 10,  11  0 24,
```

Alert level G

```
PLAT004_ALERT_5_G Polymeric Structure Found with Maximum Dimension          1 Info
PLAT083_ALERT_2_G SHELXL Second Parameter in WGHT  Unusually Large      18.98 Why ?
PLAT142_ALERT_4_G s.u. on b - Axis Small or Missing .............    0.00010 Ang.
PLAT143_ALERT_4_G s.u. on c - Axis Small or Missing .............    0.00030 Ang.
PLAT242_ALERT_2_G Low    'MainMol' Ueq as Compared to Neighbors of       C01O Check
PLAT242_ALERT_2_G Low    'MainMol' Ueq as Compared to Neighbors of       C022 Check
PLAT242_ALERT_2_G Low    'MainMol' Ueq as Compared to Neighbors of       C029 Check
PLAT242_ALERT_2_G Low    'MainMol' Ueq as Compared to Neighbors of       C02A Check
PLAT242_ALERT_2_G Low    'MainMol' Ueq as Compared to Neighbors of       C02D Check
PLAT242_ALERT_2_G Low    'MainMol' Ueq as Compared to Neighbors of       C02H Check
PLAT242_ALERT_2_G Low    'MainMol' Ueq as Compared to Neighbors of       C02N Check
PLAT242_ALERT_2_G Low    'MainMol' Ueq as Compared to Neighbors of       C02O Check
PLAT242_ALERT_2_G Low    'MainMol' Ueq as Compared to Neighbors of       C02Q Check
PLAT242_ALERT_2_G Low    'MainMol' Ueq as Compared to Neighbors of       C02S Check
PLAT242_ALERT_2_G Low    'MainMol' Ueq as Compared to Neighbors of       C02X Check
PLAT242_ALERT_2_G Low    'MainMol' Ueq as Compared to Neighbors of       C02Y Check
PLAT434_ALERT_2_G Short Inter HL..HL Contact F00U     ..F01I     .       2.71 Ang.
                                              -1+x,y,z  =      1_455 Check
PLAT720_ALERT_4_G Number of Unusual/Non-Standard Labels ..........        136 Note
              Ag01    Ag02    Ag03    Ag04    Ag05    Ag06    F007    F008
```

```
              F009    F00A    F00B    O00C    F00D    F00E    O00F    F00G
              F00H    F00I    F00J    F00K    F00L    F00M    N00N    F00O
              F00P    F00Q    F00R    F00S    F00T    F00U    F00V    F00W
              F00X    N00Y    O00Z    F010    F011    F012    F013    F014
              F015    N016    N017    N018    N019    N01A    F01B    N01C
              N01D    C01E    H01A    H01B    N01F    N01G    F01H    F01I
              F01J    F01K    C01L    H01C    H01D    N01M    C01N    C01O
              F01P    C01Q    C01R    H01R    C01S    C01T    H01T    C01U
              H01E    H01F    C01V    H01G    H01H    C01W    H01W    C01X
              H01X    C01Y    C01Z    C020    C021    C022    C023    C024
              H024    C025    C026    C027    H027    C028    C029    C02A
              C02B    H02A    H02B    H02C    C02C    C02D    C02E    H02D
              H02E    C02F    C02G    C02H    C02I    C02J    H02J    C02K
              H02K    C02L    H02O    C02M    C02N    C02O    C02P    C02Q
              C02R    H02R    C02S    C02T    C02U    H02F    H02G    C02V
              H02H    H02I    H02L    C02W    H02M    H02N    C02X    C02Y
PLAT802_ALERT_4_G CIF Input Record(s) with more than 80 Characters          1 Info
PLAT883_ALERT_1_G Absent Datum for _atom_sites_solution_primary ..     Please Do !
PLAT912_ALERT_4_G Missing # of FCF Reflections Above STh/L=  0.600        206 Note
PLAT933_ALERT_2_G Number of HKL-OMIT Records in Embedded .res File          7 Note
               -7 12 23,  -6 12 26,  -6 13 19,  -6 13 20,  -5 13 26,  -5 14 20,
               -5 14 21,
PLAT941_ALERT_3_G Average HKL Measurement Multiplicity ..........        4.8 Low
PLAT969_ALERT_5_G The 'Henn et al.' R-Factor-gap value ..........      2.476 Note
              Predicted wR2: Based on SigI**2  3.73 or SHELX Weight  8.17
PLAT978_ALERT_2_G Number C-C Bonds with Positive Residual Density.          0 Info
```

```
   0 ALERT level A = Most likely a serious problem - resolve or explain
   1 ALERT level B = A potentially serious problem, consider carefully
   5 ALERT level C = Check. Ensure it is not caused by an omission or oversight
  25 ALERT level G = General information/check it is not something unexpected

   3 ALERT type 1 CIF construction/syntax error, inconsistent or missing data
  17 ALERT type 2 Indicator that the structure model may be wrong or deficient
   4 ALERT type 3 Indicator that the structure quality may be low
   5 ALERT type 4 Improvement, methodology, query or suggestion
   2 ALERT type 5 Informative message, check
```

Validation response form

Please find below a validation response form (VRF) that can be filled in and pasted into your CIF.

```
# start Validation Reply Form
_vrf_PLAT042_lcz068
;
PROBLEM: Calc. and Reported MoietyFormula Strings  Differ     Please Check
RESPONSE: ...
;
_vrf_PLAT090_lcz068
;
PROBLEM: Poor Data / Parameter Ratio (Zmax > 18) ........       7.64 Note
RESPONSE: ...
;
_vrf_PLAT213_lcz068
;
```

```
PROBLEM: Atom F01K            has ADP max/min Ratio .....        3.1 prolat
RESPONSE: ...
;
_vrf_PLAT342_lcz068
;
PROBLEM: Low Bond Precision on  C-C Bonds ...............    0.01296 Ang.
RESPONSE: ...
;
_vrf_PLAT911_lcz068
;
PROBLEM: Missing FCF Refl Between Thmin & STh/L=    0.600          3 Report
RESPONSE: ...
;
# end Validation Reply Form
```

It is advisable to attempt to resolve as many as possible of the alerts in all categories. Often the minor alerts point to easily fixed oversights, errors and omissions in your CIF or refinement strategy, so attention to these fine details can be worthwhile. It is up to the individual to critically assess their own results and, if necessary, seek expert advice.

PLATON version of 17/09/2025; check.def file version of 17/09/2025

---

duplicate check

No duplication found

Datablock lcz068 - ellipsoid plot

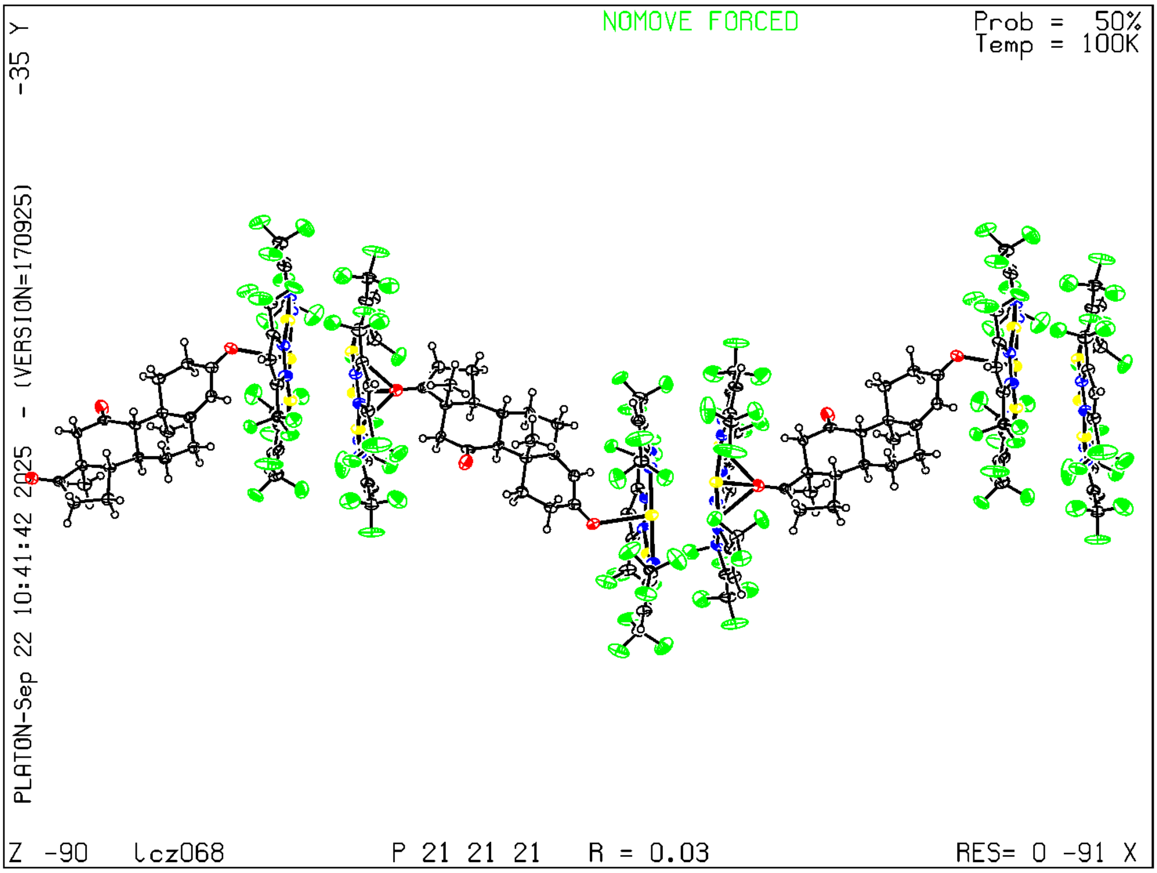

Supplement: Supplementary file 2 — Supporting File 2: anie72462‐sup‐0002‐DataFile.zip. [file ANIE-65-e1218503-s002.zip › supplementary_materials_crystallographic_data submit.zip!/cif_and_checkcif(1-114)/Ag3Pz3_68/Ag3Pz3_68_checkcif.pdf]
